# The Red Fox Y-Chromosome in Comparative Context

**DOI:** 10.3390/genes10060409

**Published:** 2019-05-28

**Authors:** Halie M. Rando, William H. Wadlington, Jennifer L. Johnson, Jeremy T. Stutchman, Lyudmila N. Trut, Marta Farré, Anna V. Kukekova

**Affiliations:** 1 Illinois Informatics Institute, University of Illinois at Urbana-Champaign, Urbana, IL 61801, USA; 2 Department of Animal Sciences, College of Agricultural, Consumer and Environmental Sciences, University of Illinois at Urbana-Champaign, Urbana, IL 61801, USA; jjohnso@illinois.edu (J.L.J.); stutchm2@illinois.edu (J.T.S.); avk@illinois.edu (A.V.K.); 3 Tropical Research and Education Center, Agronomy Department, University of Florida, Homestead, FL 33031, USA; wwadlington@ufl.edu; 4 Institute of Cytology and Genetics of the Siberian Branch of the Russian Academy of Sciences, Novosibirsk 630090, Russia; trut@bionet.nsc.ru; 5 School of Biosciences, University of Kent, Canterbury, Kent CT2 7NJ, UK; m.farre-belmonte@kent.ac.uk

**Keywords:** Y-chromosome, carnivore, *Vulpes vulpes*, sex chromosomes, MSY, Y-chromosome genes, copy-number variation, *BCORY2*, *UBE1Y*, next-generation sequencing

## Abstract

While the number of mammalian genome assemblies has proliferated, Y-chromosome assemblies have lagged behind. This discrepancy is caused by biological features of the Y-chromosome, such as its high repeat content, that present challenges to assembly with short-read, next-generation sequencing technologies. Partial Y-chromosome assemblies have been developed for the cat (*Felis catus*), dog (*Canis lupus familiaris*), and grey wolf (*Canis lupus lupus*), providing the opportunity to examine the red fox (*Vulpes vulpes*) Y-chromosome in the context of closely related species. Here we present a data-driven approach to identifying Y-chromosome sequence among the scaffolds that comprise the short-read assembled red fox genome. First, scaffolds containing genes found on the Y-chromosomes of cats, dogs, and wolves were identified. Next, analysis of the resequenced genomes of 15 male and 15 female foxes revealed scaffolds containing male-specific *k*-mers and patterns of inter-sex copy number variation consistent with the heterogametic chromosome. Analyzing variation across these two metrics revealed 171 scaffolds containing 3.37 Mbp of putative Y-chromosome sequence. The gene content of these scaffolds is consistent overall with that of the Y-chromosome in other carnivore species, though the red fox Y-chromosome carries more copies of *BCORY2* and *UBE1Y* than has been reported in related species and fewer copies of *SRY* than in other canids. The assignment of these scaffolds to the Y-chromosome serves to further characterize the content of the red fox draft genome while providing resources for future analyses of canid Y-chromosome evolution.

## 1. Introduction

Over the last decade, the emergence of next-generation sequencing (NGS) technologies has catalyzed a proliferation of reference assemblies, including those of non-traditional model species (e.g., [1]). However, only a small subset of such assemblies includes the Y-chromosome. This disparity is driven by the challenges associated with assembling Y-chromosome sequence, especially in eutherian mammals. One of the main issues is that the Y-chromosome contains a high proportion of repetitive sequences, which are difficult to assemble from short sequencing reads [2,3,4,5]. Sequencing projects specifically targeting the Y-chromosome often circumvent this problem using traditional methods, such as bacterial artificial chromosome (BAC) cloning or long-read Sanger sequencing technology [6,7,8], but this dependence on more expensive technologies means that many of the advances made towards a reduction in the financial and time investment required for genome assembly do not extend to Y-chromosome assembly. While approaches to de novo assembly that utilize third-generation long-read sequencing technologies are emerging (e.g., [9]), these approaches remain largely inaccessible to assembly projects targeting non-model species.

Work in several species has indicated that Y-chromosome information can be extracted from genomes sequenced with short-read technologies. De novo contigs constituting a partial assembly (186 Kbp) of the horse Y-chromosome were assembled using Roche 454 reads to conduct targeted resequencing of horse BAC clones that had been selected based on homology to Y-chromosome genes in other mammals [10]. In the tongue sole, which is a flatfish with a 477-Mbp genome, scaffolds in the reference genome that corresponded to the constitutively haploid chromosome (W) were identified by sequencing the genomes of a homogametic (ZZ) and a heterogametic (ZW) fish at 212× coverage and by comparing the depth of coverage across the scaffolds between the male and female individuals [11]. Using a similar approach, the 72,214 scaffolds comprising the Illumina-sequenced 2.3-Gbp polar bear reference genome [12] were analyzed by comparing male-to-female depth of coverage across scaffolds and identifying scaffolds syntenic to Y-chromosome genes found in other eutherian mammals (human, dog, chimpanzee, and mouse) [13]. This analysis identified 1.9 Mbp of putative Y-chromosome sequence across 112 scaffolds of the polar bear reference genome. In another de novo carnivore assembly, the grey wolf, comparison of male and female sequence coverage alongside known canine Y-linked genes similarly allowed for the identification of putative Y-chromosome scaffolds [14]. These studies indicate that when a heterogametic individual is sequenced for de novo reference assembly, even when the assembly project uses short-read NGS technology, fragments (contigs and/or scaffolds) containing identifiable fragments of the constitutively haploid chromosome sequence are often produced.

The red fox (*Vulpes vulpes*) is a non-traditional mammalian genomic model in which characterization of Y-chromosome diversity is of particular interest. The red fox is the world’s widest-spread terrestrial carnivore [15,16], and the species’ behavioral ecology results in males dispersing more widely than females [17]. Mitochondrial DNA (mtDNA) haplotypes have been characterized in a number of populations to address a range of questions related to red fox population history and diversity (e.g., [18,19,20,21,22,23,24]). However, because mtDNA is matrilineally inherited, mtDNA diversity alone may not accurately reflect genome-wide diversity [25,26]. Prior to the assembly of a red fox reference genome [27], Y-chromosomal resources for the red fox were limited to two dog-derived microsatellite markers [18]. 

Opportunities to develop Y-chromosome resources for the red fox expanded with the recent red fox genome sequencing project [27], which produced 2.5 Gbp of sequence from a farm-bred male fox. The draft genome is organized in 676,878 scaffolds ranging in size from as large as 55.7 Mb to as small as 100 bp, with a scaffold N50 of 11.8 Mbp [27]. Preliminary analysis [28] of two scaffolds found to show higher synteny with the dog Y-chromosome than any other dog chromosome facilitated the development of 11 novel male-specific microsatellite markers that were used to conduct a preliminary analysis of patterns of diversity across red fox populations. Although the development of genomic resources for the red fox has focused primarily on experimentally bred tame and aggressive lines developed and maintained through the Russian Farm Fox Experiment at the Institute for Cytology and Genetics in Novosibirsk, Russia [29], the preliminary analysis of inter-population diversity using the 13 microsatellite markers suggested that resources developed in farm-bred foxes are still useful for ascertaining variation in geographically diverse populations [28].

The development of additional Y-chromosome resources for the red fox remains a priority. In particular, Y-chromosomal single nucleotide polymorphism (SNP) markers would provide higher resolution than microsatellite markers and allow for phylogenetic comparisons over longer timescales [30]. Identifying the sequence of the red fox Y-chromosome would represent a significant step towards a dense SNP marker set for these analyses. The two known Y-chromosome scaffolds comprise only 1 Mbp of sequence, whereas the male-specific region of the Y-chromosome (MSY) is approximately 2.5 Mbp in many other carnivores [6], suggesting that additional sequence may be present in the assembly. Likewise, the two Y-scaffolds contain only nine predicted genes [27,28], including only four of the 11 MSY genes consistently observed across carnivore species [31]. Therefore, additional analysis is required to identify Y-chromosome sequence, including genes, present in the red fox draft genome. 

The present analysis characterizes MSY sequence using three complementary approaches: analysis of gene content within scaffolds, identification of male-specific sequence motifs, and comparison of sequencing depth between males and females. The first approach, similarity between the scaffold sequence content and known Y-chromosome genes, has been used to identify MSY sequence in other mammals (e.g., [10,13,14,32]). MSY assemblies for two species closely related to the red fox, the cat (*Felis catus;* KP081775.1) and dog (*Canis lupus familiaris;* KP081776.1), are available [6], with the red fox’s least common ancestors (LCA) with the cat estimated at 50–65 million years ago (MYA) and with the dog estimated at 9–15 MYA [33,34]. Many dog and cat Y-chromosome genes and protein sequences have been deposited in the databases maintained by the National Center for Biotechnology Information (NCBI) [7,35]. These sequences can therefore be used as probes to identify scaffolds in the draft genome that are likely to contain MSY sequence.

Two additional methods are used to complement the syntenic analysis. These methods are not restricted to regions that contain genes, but instead examine sex-specific patterns in whole genome resequencing data (WGS) mapped onto the reference assembly. Specifically, 15 male and 15 female red foxes bred on the same farm as the reference genome donor fox were resequenced at a depth of 2.5× per fox [27]. The WGS data were analyzed to identify scaffolds likely to contain MSY sequence based on two metrics: sequence motifs exclusive to males and therefore likely to be derived from the MSY, and differences in sequencing depth in the heterogametic (male) versus homogametic (female) individuals. For the first metric, Carvalho and Clark [36] developed software to identify male-specific sequence by fragmenting the scaffolds into *k*-mers and tabulating *k*-mer frequency in the male and female resequencing data. For the second, copy number variation (CNV) was characterized with CNV-seq [37] to identify differences in sequence coverage of the scaffolds in male and female sequencing data. Analyzing the scaffolds along these two axes facilitates the identification of the scaffolds most likely to contain Y-chromosome sequence and thus provides an approach to identify MSY sequence bioinformatically. Used together, these approaches represent a consilience-oriented approach to the identification of MSY sequence from fragments assembled with short-read NGS technologies.

## 2. Materials and Methods 

First, we sought to identify red fox orthologs of genes located on the MSY of dog and cat, which are two carnivores closely related to the red fox [6,7,35,38] (Table 1). Most of these genes are X-degenerate, meaning they are thought to be derived from genes shared by the X- and Y-chromosomes in their ancestral state as a pair of homologous autosomes [39], but some (e.g., *TETY2* or *FLJ36031Y*) were more recently transposed to the Y-chromosome from the X-chromosome or an autosome [6,7,35] (Table 1). Dog protein sequences or transcripts were downloaded, as available, from the NCBI Sequence Read Archive (SRA) for each of the genes on the dog MSY. *DYNG*, which is a novel Y-chromosomal gene identified in dog [6], was excluded at this stage due to the lack of a protein or mRNA sequence in NCBI SRA or other databases. Cat transcripts or nucleotide sequences were downloaded, as available, for the four genes present on the feline, but not the canine, MSY [6] (*AMELY*, *FLJ36031Y*, *RPS4Y*, and *TETY1*) and for *EIF2S3Y*, whose canine protein sequence has not been deposited. 

Gene sequences were then mapped against all scaffolds longer than 5 Kbp in the draft red fox genome [27] using translated BLAST (tblastn) or standard nucleotide BLAST (blastn), as appropriate, in the command line implementation of BLAST+ version 2.2.29 [40]. A minimum e-value of 10^−5^ was specified. Hits to autosomes and the X-chromosome were removed based on the chromosomal positions assigned to the scaffolds [41] (Table S1). Of the remaining hits, the best hit was determined to be the scaffold with the longest continuous stretch of query sequence mapping with greater than 90% (canine) or 80% (cat) identity. Hits from multiple scaffolds were included as long as each scaffold contained at least one hit meeting the percent identity criteria.

Next, the scaffolds matching one or more known carnivore MSY genes were examined to identify whether they also contained any predicted genes from the red fox draft annotation [27]. Predicted gene sequences from the annotation that had been translated into protein sequences [27] were compared to *C. l. familiaris* sequences deposited in NCBI using the web browser version of tblastn. The best match was selected based on total score. When the best dog hit had a known MSY homolog, the positions of the dog-vs-fox and fox-vs-dog queries were compared to determine whether they overlapped.

Additionally, a recent analysis of the wolf (*Canis lupus lupus*) Y-chromosome [14] identified and provided reference positions for three genes not previously reported in dogs or cats (*TMSB4Y*, *AP1S2Y*, and *WWC3Y*) along with a wolf ortholog of the dog gene *DYNG* (Table 2). The protein sequences of the dog X-chromosome genes paralogous to *AP1S2Y* and *WWC3Y* were downloaded from NCBI. For *TMSB4Y* and *DYNG*, the nucleotide sequence of the corresponding region was extracted from the wolf reference genome assembly [42] using the approximate positions reported [14] and compared to the red fox genome using blastn. Because the genes *AMELY*, *FLJ36031Y*, and *RPS4Y* were not analyzed in the grey wolf Y-chromosome assembly [14], the cat sequences of these genes were also compared to the grey wolf reference genome [42] to evaluate whether these genes were present on wolf Y-linked scaffolds (Table S2).

**Table 1:** The 22 genes of interest for the fox male-specific region of the Y-chromosome (MSY) based on cat and dog. Genes were selected as probes based on their presence on the MSY of dogs and/or cats [6,7,43]. Where a gene is present in one species and absent in the other, grey shading is used to highlight the derived state relative to other carnivores. The term ‘X-transposed’ denotes that *OFD1Y* may have been recently transposed from the X to the Y chromosome [44]. Evolutionary origins of genes in carnivores are based on analyses of the dog and cat Y-chromosomes [6,7,35]. The gene *TXLNGY* was previously called *CYorf15*, and *UBE1Y* is also referred to as *UBA1Y* in the literature.

Gene/Gene Family	Dog	Cat	Sequence Used	Sequence Species	Origin
*AMELY*	-	+	EU879968	Cat	X-degenerate
*BCORY1*	+	-	AGS47779	Dog	X-degenerate
*BCORY2*	+	-	AGS47770	Dog	X-degenerate
*CUL4BY*	+	+	AGS47784	Dog	X-degenerate
*DDX3Y*	+	+	JX964855	Dog	X-degenerate
*EIF2S3Y*	+	+	EU879975	Cat	X-degenerate
*EIF1AY*	+	+	AKI82173	Dog	X-degenerate
*FLJ36031Y*	-	+	NP_001108352	Cat	Ampliconic (autosome-derived)
*HSFY*	+	+	AKI82172	Dog	Ampliconic (X-derived)
*KDM5D*	+	+	AGS47774.1	Dog	X-degenerate
*OFD1Y*	+	+	AGS47782.1	Dog	X-transposed
*RPS4Y*	-	+	EU879986	Cat	X-degenerate
*RBMYL*	+	-	AKI82176	Dog	X-degenerate
*SRY*	+	+	AAD40225	Dog	X-degenerate
*TETY1*	-	+	AZD12964.1	Cat	Ampliconic (autosome-derived)
*TETY2*	+	+	AGS47775	Dog	Ampliconic (X-derived)
*TSPY*	+	+	AGS47785	Dog	Ampliconic (X-derived)
*TXLNGY*	+	+	AKI82175.1	Dog	Ampliconic (X-derived)
*UBE1Y*	+	+	AKI82178	Dog	X-degenerate
*USP9Y*	+	+	AKI82171	Dog	X-degenerate
*UTY*	+	+	NM_001284484	Dog	X-degenerate
*ZFY*	+	+	JX964866	Dog	X-degenerate

In order to identify the position of the pseudoautosomal boundary in the red fox, whole genome resequencing reads from 30 farm-bred red foxes—drawn equally from each of three lines maintained at the Institute for Cytology and Genetics in Novosibirsk, Russia (NCBI BioProject PRJNA376561; [27])—were aligned using Bowtie2 [45] to a version of the dog reference genome assembly that was created by concatenating canFam3.1 [46] and the dog Y-chromosome assembly (GenBank: KP081776.1; [6]). Of these 30 foxes, 15 were male and 15 were female, and each fox was sequenced at approximately 2.5× coverage [27]. Duplicates were marked at the level of the individual with Picard MarkDuplicates [47] and then the alignments were pooled at the population level (experimental line) and recalibrated with RealignerTargetCreator and IndelRealigner in the Genome Analysis Toolkit version 3.7 [48]. Data were combined across lines for all individuals of each sex, and depths were then tabulated for males and females separately using SAMTools depth [49] for the X-chromosome only (-r chrX). Average depth was calculated for each sex in windows of 100 Kbp and 1 Mbp in Python 2.7 and then plotted in R [50] with ggplot2 [51]. 

Male-specific sequence motifs were then identified using a pipeline for comparing *k*-mers across two groups [36]. The pipeline in the Full Methods section of [36] was followed to prepare the red fox reference genome version 2.2 [27], which had been masked using RepeatMasker 4.0.5 [52] with the carnivore repeat library, for analysis with the script YGS.pl described in [36]. YGS.pl was then used to compare the male and female 18-mers to identify those that were single-copy (i.e., only one copy present in the reference genome), valid (i.e., found in the male sequencing reads) and unmatched (i.e., found in the male but not the female sequencing reads). In order to reduce computing time during this analysis, only scaffolds 1 Kbp or longer were analyzed. These 12,625 scaffolds comprise 96.1% of the complete draft genome sequence by length. Scaffolds found to have no valid single-copy *k*-mers in the YGS.pl output were excluded from downstream analysis. The percent V_SC_UK (valid, single-copy k-mers unmatched in females) on each scaffold was normalized by calculating the standard score (i.e., by subtracting the mean and dividing by the standard deviation as estimated across all scaffolds).

In order to compare sequencing depth across the red fox draft genome between males and females, the male and female reads were aligned using Bowtie2 [45] to the 676,878 scaffolds of the repeat-masked red fox reference genome, as described above. The bam files corresponding to the aligned reads from each individual were pooled by sex for downstream analysis. The overall depth of coverage was estimated for the males and females using SAMTools depth [49]. CNV were analyzed using CNV-Seq [37] to identify differences in depth-of-coverage along the genome in the male and female resequencing data. CNV-Seq was run with the genome size set to 2,496,140,267 bp and the window size to 10,000 bp. The female data was used as the reference and the male data as the test data. CNV-Seq estimated the number of reads mapping to each 10,000-bp window along each masked scaffold, with 5000 bp of overlap between windows. Any window containing fewer than 100 reads, which corresponded to a coverage of less than 0.01×, was excluded from downstream analysis. For each window, the percentage of mapped reads that originated in the male resequencing data was estimated by dividing the number of reads mapping to the window in the male dataset by the total number of reads mapping to the window across both datasets. The percentages were again normalized to a standard score. All scaffolds shorter than 1 Kbp were dropped from the CNV-Seq output, as they been excluded from the analysis with YGS.pl. 

The scores corresponding to each window from CNV-Seq (sex-based depth) and from YGS.pl (male-specificity of 18-mers) were plotted, first, for only the windows on scaffolds with *a priori* chromosomal origins assigned [27,28,41], and then for all windows. Given that scaffolds containing sequence from the X-chromosome, autosomes, and the Y-chromosome were expected to form distinct clusters, we applied the *k*-means clustering algorithm [53] to the data with *k*, or the number of centers, set to 3. *k*-means clustering is an unsupervised machine learning algorithm that assigns individual data points to one of *k* clusters and adjusts the positions of the centers of the clusters to minimize the point-to-center distance across all points. The particular implementation used here was R’s native *k*-means clustering function [54]. Clustering was conducted on a matrix containing, for each window, the standardized percent of reads mapping to the window that came from the male resequencing data (as identified with CNV-Seq) and the standardized percent of *k*-mers on the scaffold that were valid, single-copy, and unmatched in the female reads (as identified with YGS.pl). The maximum number of sets of random centers to be selected (nstart) was set to 100. 

The clusters identified by *k*-means clustering were then evaluated to determine how likely they were to represent the three expected classes of chromosomes. Some scaffolds had been previously assigned to a position on the X-chromosome or autosomes [41] or identified as likely to contain Y-chromosome sequence (Table 3; [27,28]). These scaffolds were used to examine whether the clusters consistently contained scaffolds with the same chromosomal origin. The number of windows assigned to each cluster was also tabulated for each scaffold. If more than 15% but less than 85% of the windows on a scaffold were assigned to a cluster, the scaffold was evaluated manually. 

The scaffolds assigned to the Y-chromosome using this process were then examined to ensure that all metrics were consistent with what was expected for the Y-chromosome. SAMTools depth was used to estimate the sequencing depth along the putative Y-chromosome scaffolds for the male and female WGS reads mapped to the fox reference assembly. Depth along each scaffold was visualized with ggplot2, as described above, in windows of 5 Kbp or 10% of scaffold length, whichever was smaller.

The next step was to reassemble the putative male-specific sequence using a different alignment algorithm than was used in the red fox reference genome assembly project. The alignment of the male resequencing data to the fox genome, as described above, was filtered using SAMTools view with the -L parameter to extract only the reads that mapped to putative Y-scaffolds. The 15 libraries from the genome project [27], which provide 93.9× coverage from a single male fox (BioProject PRJNA378561), were then aligned to the original draft genome (vv2.2; [27]) with the program Burrows-Wheeler Aligner (BWA) [55]. The alignments were cleaned with SAMTools fixmate [49] and then the reads mapping to the putative Y-chromosome scaffolds were extracted from each alignment using SAMTools view with the -L parameter. All of the alignments were then sorted by read name (using the -n parameter) with SAMTools sort and extracted into paired end fastq files using BEDTools bamToFastq [56]. Each of the fastq files containing paired-end reads from the genome project was cleaned to remove duplicate reads using the functions dedupe and reformat from BBMap version 38.35 [57]. 

ABySS version 2.1.5 [58,59] was then used to assemble the reads, with the paired-end reads from the genome assembly project and pooled male WGS data provided for assembly of the contigs (lib) and the mate-pair libraries from the genome project for scaffolding contigs (mp). Per the ABySS 2.0 manual, the program was tested with values of *k* (*k*-mer size) to optimize for N50 and assembly size. The values of *k* tested ranged from 50 to 96.

The ABySS assembly and putative Y-chromosome draft genome scaffolds were then compared: first, to each other (percent identity = 95%; filtering = one-to-one); then to the dog Y-chromosome assembly (percent identity = 90%; filtering = one-to-one); and finally to the cat Y-chromosome assembly (percent identity = 80%; filtering = one-to-one) using MashMap [60]. The results of the inter-assembly alignments were visualized with MashMap’s visualization script, generateDotPlot. MashMap’s output was also visualized in Evolution Highway [61]. 

## 3. Results

### 3.1. Gene Analysis

Based on the BLAST results, 22 out of the 25 genes previously identified on the dog, wolf, and/or cat MSY were assigned to a position on one or more red fox scaffold(s) not previously assigned to an autosome or the X-chromosome (Table 3). The genes that were not identified were two cat MSY genes (*TETY1* and *FLJ36031Y*) that have not been previously reported in canids and *TSPY*, which has been reported to occur in multiple copies on the MSY of cat, dog, and wolf [6,7,14,35]. BLAST results were also examined to determine whether any expected X-chromosomal or autosomal paralogs were identified (Table S1). Twelve genes could be assigned to a single position on a putative MSY scaffold, and in five additional cases, a candidate MSY copy of a gene was identified but was split across multiple scaffolds (Table 3). In total, 22 scaffolds of 5 Kbp or longer were found to contain an MSY gene or gene fragment. Putative Y-chromosome orthologs were identified for all 11 core carnivore MSY genes [31].

Orthologs were identified for 17 out of the 18 genes on the dog MSY, corresponding to the 11 core carnivore MSY genes and six additional genes. No match to the protein sequence of dog *TSPY* was identified in the red fox scaffolds. Two scaffolds contained sequence similar to the dog *BCOR*-derived MSY genes (*BCORY1* and *BCORY2*). The BLAST results indicated that scaffold310 contained one copy and scaffold360 the other, with most exons mapping to both scaffolds. A blastn search of the NCBI’s online repository indicated that *BCORY2* is more similar to the sequence of scaffold310 and *BCORY1* to scaffold360 (Appendix A). The data also suggest that two of the four genes previously identified on the cat MSY but not on that of dog are also present on the red fox MSY. *RPS4Y* mapped to scaffold292, which was previously validated as containing male-specific sequence [28]. *AMELY* was split across scaffold7085 and scaffold549, with an additional copy identified on an X-linked scaffold (Table S1). Candidate orthologs of two genes recently identified on the wolf MSY, *AP1S2*, and *TMS4BY*, were also identified in the red fox scaffolds.

Four of the scaffolds that were matches for dog MSY gene queries (Table 3) also contained predicted genes [27] (Table 4). For eight of these 11 predicted fox genes, a BLAST query of NCBI’s nucleotide database identified a known dog MSY gene (Table 4) that had been assigned to an overlapping position on the scaffold by tblastn query against the red fox reference genome (Table 3). Additionally, for Vulp_V012195, which overlaps the estimated position of *EIF2S3Y* (Table 3), the closest hit in NCBI’s database was the dog gene *EIF2S3*, which is on the X-chromosome. This result is consistent with expectations, given that the canine *EIF2S3Y* sequence has not been deposited in NCBI’s nucleotide database. Vulp_V012196 most closely matched *WWC3*, a dog X-chromosome gene that is located near the pseudoautosomal boundary in carnivores [6] and for which a Y-chromosome paralog was recently reported in the grey wolf and named *WWC3Y* [14].

Vulp_V011273 on scaffold292 did not match a dog gene with a known homolog on the dog Y-chromosome. Vulp_V011273 was predicted using a transcript of a human autosomal gene, RPS28 (ENSP00000472469) [27]. The closest match in the NCBI tblastn search against dogs was RPS20, which is located on dog chromosome 20 (Table 4). When the NCBI search was expanded to all carnivores, the closest match was a predicted pseudogene in a female fur seal (XR_003206562.1). The fox predicted gene matched with 97% identity to the dog Y-chromosome assembly (GenBank: KP081776.1) at 224,427–224,621 bp, suggesting a high degree of synteny in this region between the two species. 

### 3.2. Pseudoautosomal Boundary Estimation

**Figure 1:**
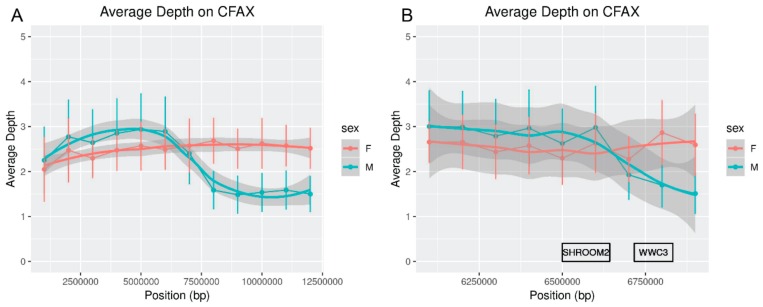
Average per-fox depth in males and females along dog chromosome X (CFAX). Bars represent the standard deviation of mean estimates, while the grey shading indicates the confidence interval based on smoothing with geom_smooth() in ggplot2 [51]. (**A**) Averages were calculated in 1-Mbp intervals. (**B**) Averages were calculated in 100-Kbp windows and are shown only for the region from chrX 6 Mbp to 7 Mbp. The two genes flanking the pseudoautosomal boundary on CFAX, *SHROOM2* (pseudoautosomal) and *WWC3* (X-chromosomal), are indicated as rectangles along the *x*-axis.

Comparing the depth of sequencing in males and females along the dog X-chromosome indicated that the red fox pseudoautosomal boundary is likely adjacent to the gene *SHROOM2*, consistent with findings in other carnivores [6,62]. Males and female foxes show similar average depths over the first 6 Mbp of X-chromosome sequence (Figure 1). *SHROOM2* is located at approximately chrX: 6,642,728–6,499,660 bp in dogs and corresponds to the drop in male sequence mapping depth (Figure 1). 

### 3.3. Identification of Male-Specific k-mers

**Figure 2:**
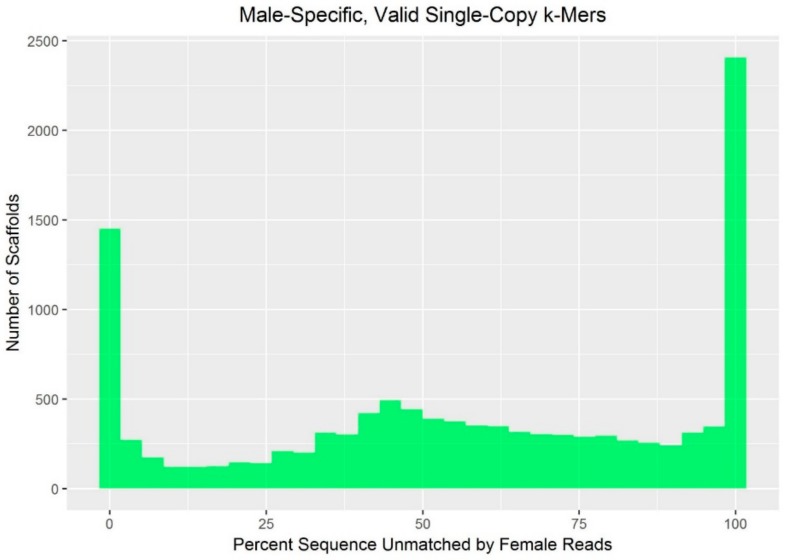
The distribution of male-specific sequence among scaffolds. This histogram indicates the percent of valid, single-copy *k*-mers unmatched in the female reads across all scaffolds evaluated.

Valid single-copy (VSC) 18-mers were identified on 10,522 of the 12,625 largest scaffolds, and the number of 18-mers on each scaffold that were unmatched by the female reads (VSC_UK) ranged from 0 to 15,900,208. Across these scaffolds, male-specific 18-mers (VSC_UK) were, on average, 57.1% (sd: 34.8) of all valid, single-copy 18-mers (VSC). As the proportion of *k*-mers on a scaffold unmatched in female sequencing reads approaches 100%, the scaffold is more likely to contain MSY sequence. The proportion of *k*-mers on autosomal and X-chromosomal scaffolds unmatched in female sequencing reads would be expected to be approximately 0%. In the present analysis, this distribution was multimodal (Figure 2), with less clear bimodality than reported in drosophila and humans [36]. Differences in the quality of the red fox reference assembly compared to the drosophila and human references (e.g., unfinished vs. finished; Illumina- vs. Sanger-sequenced) are likely to explain the trimodal distribution in foxes, especially because chimeric scaffolds, which are a known issue in the red fox reference assembly [27,41], introduce noise [36].

### 3.4. Copy Number Variant Analysis

In the CNV analysis, each scaffold was examined in 10-Kbp windows to compare coverage in the female (reference) versus male (test) sequencing. The average genome-wide depth of coverage from the male resequencing was 72.133 and from the female was 63.564. After removing windows with a combined coverage of less than 0.01×, an average of 52.9% of reads mapping to the remaining windows came from the male resequencing data. 

**Figure 3:**
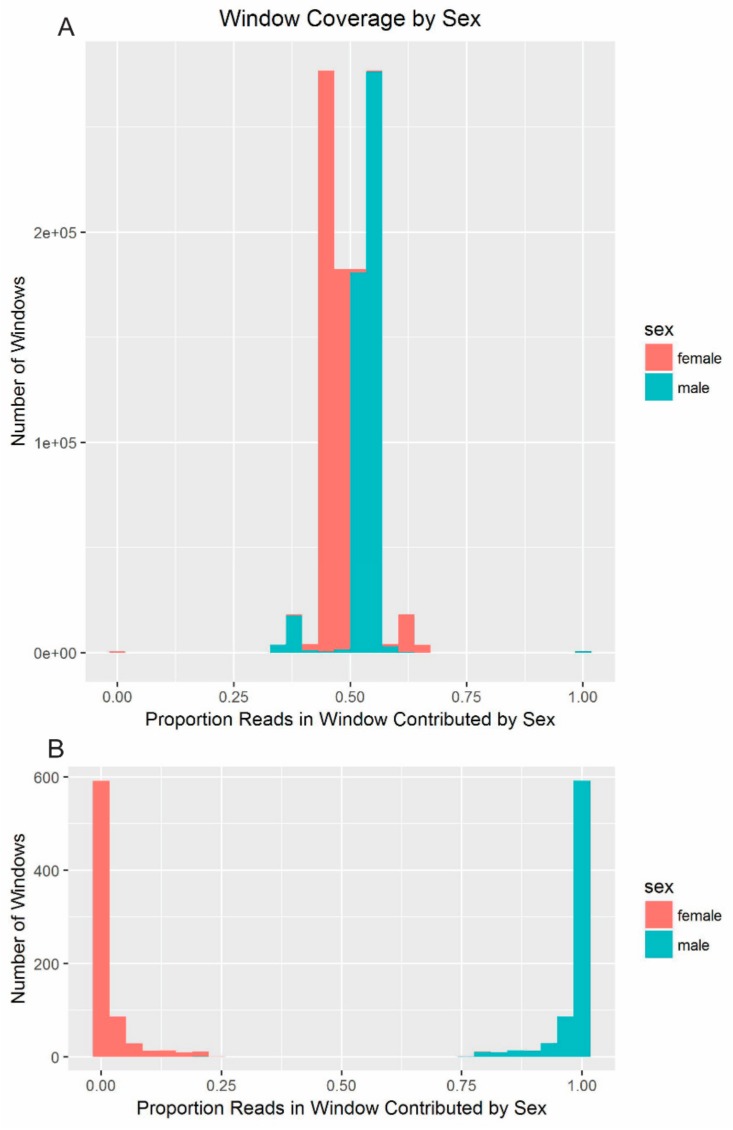
Comparison of male and female coverage of windows. (**A**) In each window, the percent of the mapped reads coming from the male and from the female resequencing data was calculated. Due to the depth of coverage in the male resequencing data being slightly higher, there is some displacement of the autosomal and X-chromosome curves off 50% and 33/67%, respectively. The expected pattern of density distribution is apparent, with males and females contributing roughly equal numbers of reads to most windows. Very small peaks are apparent at 0% and 100% that correspond to primarily male contribution. (**B**) Zoomed-in depiction of only the small peaks that represent 75% or more contribution from a single sex.

The density of the mapped reads was expected to be modulated by sex and by the ploidy of the chromosome (e.g., autosomes vs. allosomes). Because autosomes are equally represented in males and females, approximately half of the reads mapping to autosomal windows should come from males. In comparison, the male resequencing should contribute only 33% of the reads mapping to the X-chromosome because males are hemizygous. Windows containing MSY sequence were expected to have very low (~0) coverage in females, with all of the reads mapping to these regions expected to come from the male sequence. The proportion of reads mapping to each window from the female and male resequencing data supported this pattern (Figure 3).

### 3.5. Clusters of Scores

**Figure 4:**
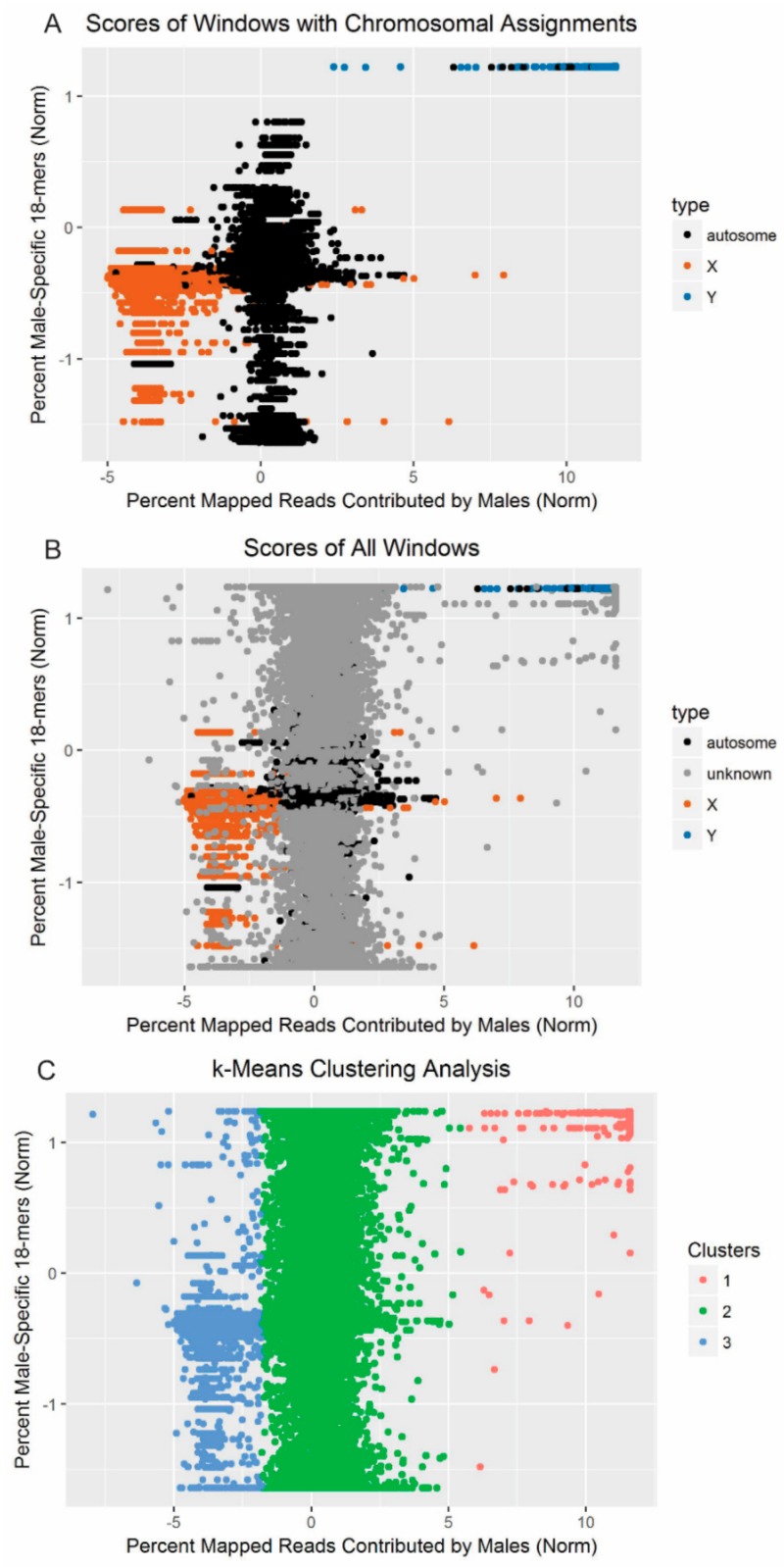
Based on copy number variation (CNV) between males and females and male-specific sequence motifs, the windows form three clusters. The percent of reads mapping to each window that originated in males (as estimated with CNV-Seq) was plotted against the percent of male-specific 18-mers comprising each scaffold (as estimated with YGS.pl). Percentages were normalized based on standard score. (**A**) Windows from scaffolds with predicted chromosomal origins are plotted, and chromosomal origin is indicated by color. (**B**) Windows from all 12,625 scaffolds analyzed are included, and chromosomal origin, when known, is indicated by color. (**C**) Each window is color-coded according to the cluster to which it was assigned by *k*-means clustering.

The CNV-Seq scores (percent mapped reads contributed by male resequencing) for each window were plotted against the YGS.pl output (percent valid single-copy *k*-mers unmatched in females). These patterns were examined in windows located on scaffolds with a known fox chromosomal position and then in all windows (Figure 4). Windows known to belong to the red fox X-chromosome, autosomes, and Y-chromosome separate out, and the overall pattern persisted when the windows with unknown chromosomal positions were added.

Running *k*-means clustering on the data revealed three clusters (Figure 4; Table 5). These clusters fit the patterns expected for sequence derived from the Y-chromosome (cluster 1), autosomes (cluster 2), and X-chromosome (cluster 3). Analysis of cluster 1 revealed that the 752 windows were located across 180 scaffolds. Of these, 176 scaffolds were at least 85% covered (by length) by windows assigned to cluster 1, corresponding to 731 windows. One scaffold (scaffold368) that fell short of this threshold at 63% was left in after manual examination. These 177 scaffolds contain 3,402,034 bp of sequence. 

Analyzing the scaffolds for which *a priori* chromosome assignments were available revealed high congruence with cluster assignment. There were 463,267 windows, corresponding to 480 scaffolds, for which predictions were available. All of the scaffolds predicted to contain Y-chromosome sequence based on previous syntenic analysis [27], gene content (Table 3; Table 4), and/or male-specific marker amplification [28] were assigned to cluster 1. One scaffold previously identified as autosomal [41] was also assigned to cluster 1. After removing cases where less than 85% of the scaffold by length had been assigned to the cluster, cluster 2 contained 344 scaffolds previously assigned to the autosomes and 1 assigned to the X-chromosome, and cluster 3 contained 50 scaffolds assigned to the X-chromosome and none assigned to the autosomes [41]. None of the other scaffolds had been assigned a position in previous analyses. 

Evaluating the windows assigned to cluster 1 also revealed that nine came from scaffolds that received low scores on the metric of male sequence uniqueness (P_VSC_UK from [36]), defined as less than 65% of the 18-mers comprising the scaffold being valid, single-copy, and unmatched in the female reads (Appendix B). These scaffolds were removed from downstream Y-chromosome analyses. After all cleaning, a total of 12 windows of the 752 assigned to the cluster (1.6%) from nine scaffolds were removed. Thus, the ABySS assembler was provided 171 scaffolds totaling 3,372,139 bp in length (Table S3).

### 3.6. Reassembly of Putative Y-Linked Sequence 

Running ABySS over a range of values of *k* (corresponding to *k*-mer size) revealed that, as expected, *k* influenced the assembly statistics (Table S4). Based on the N50 statistics and maximum scaffold size, the *k* = 66 assembly was selected for comparison to the scaffolds of the red fox draft genome assembly with MashMap. When *k* was set to 66, the fragments assembled by ABySS ranged in size from 66 bp, which was the smallest size allowed, to 256,581 bp, with an N50 of 56.4 Kbp (Table S4). None of the ABySS assemblies generated fragments as long as the 171 selected from the original genome assembly, which had a total length of 3.37 Mbp, a maximum scaffold size of 656,303 bp, and a scaffold N50 of 127.7 Kbp.

Using MashMap to align the ABySS assembly generated at *k* = 66 to the putative Y-scaffolds from the draft red fox genome revealed a high degree of concordance between the two assemblies. The alignment of the two assemblies suggests a linear relationship, despite the fact that the fragments in the ABySS assembly are shorter (Figure S1). The comparative alignments of the Y-linked red fox genome assembly scaffolds against the dog and cat MSY assemblies are available on Evolution Highway [61].

### 3.7. Gene and Segmental Replications 

**Figure 5:**
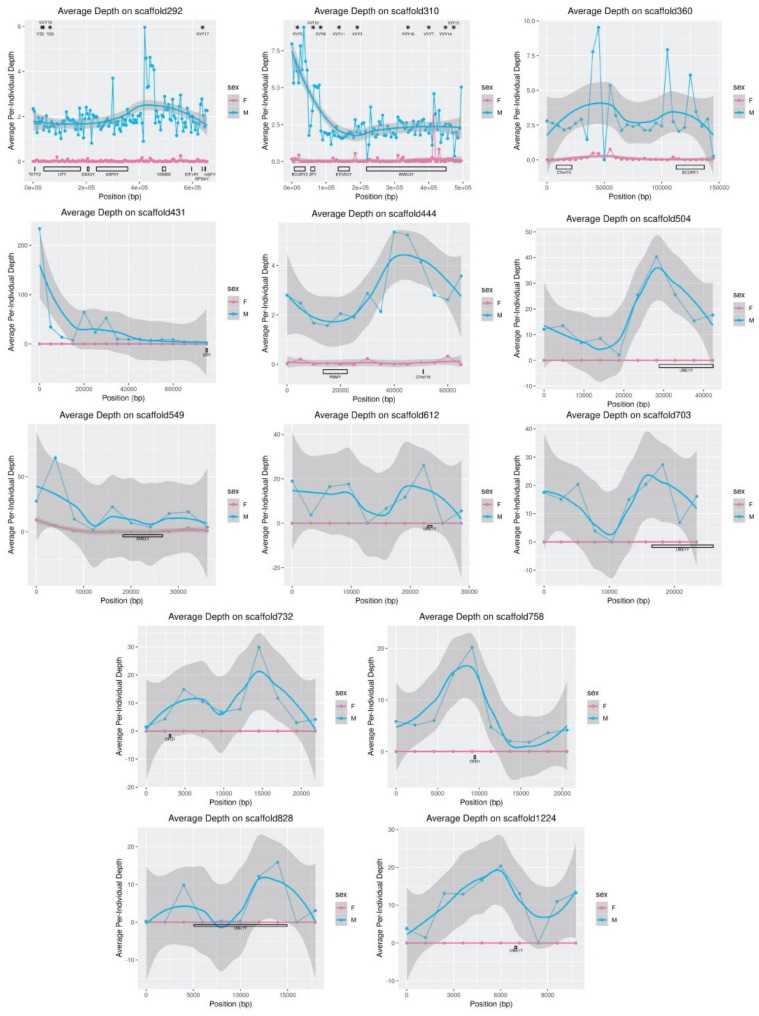
Depth of coverage of Y-scaffolds by male and female resequencing reads. Depth was averaged in males and females over 5000-bp windows for scaffolds longer than 50 Kbp, or for windows 10% of the scaffold length for shorter scaffolds. Microsatellite marker positions [28] are indicated along the top with stars; gene positions as identified in Table 3 are indicated along the bottom. Only scaffolds containing genes are shown. Axes vary depending on the observed depth (*y*) and the length of the scaffold (*x*).

Plotting the average depth of coverage along each putative MSY-linked scaffold revealed significant variation within scaffolds that was consistent with a previously hypothesized [28] segmental replication on the MSY (Figure 5). Given the overall sequence coverage in the dataset, average per-male coverage on the Y-scaffolds was expected to be approximately 2.5× in contrast to 0× for females. However, depth of coverage on scaffold310 (Figure 5) is approximately three times higher than expected in males in the region containing microsatellite marker VVY5, which was previously reported to carry up to three alleles per male despite not amplifying in females [28]. Similarly, average depth is approximately twice the expected value in the region containing microsatellite marker VVY10, for which two alleles per male were previously reported [28]. Observed depth of coverage in other regions also deviated from what was expected: for example, the gene *UBE1Y* is single-copy in cats and dogs [6], yet depth was elevated in the regions of the genome that mapped to the dog *UBE1Y* transcript. In contrast, *SRY* is likely to be present in multiple copies on the Y-chromosomes of both dogs and cats [6,35], but the region of scaffold431 containing *SRY* shows depth consistent with a single copy of the gene. 

**Figure 6:**
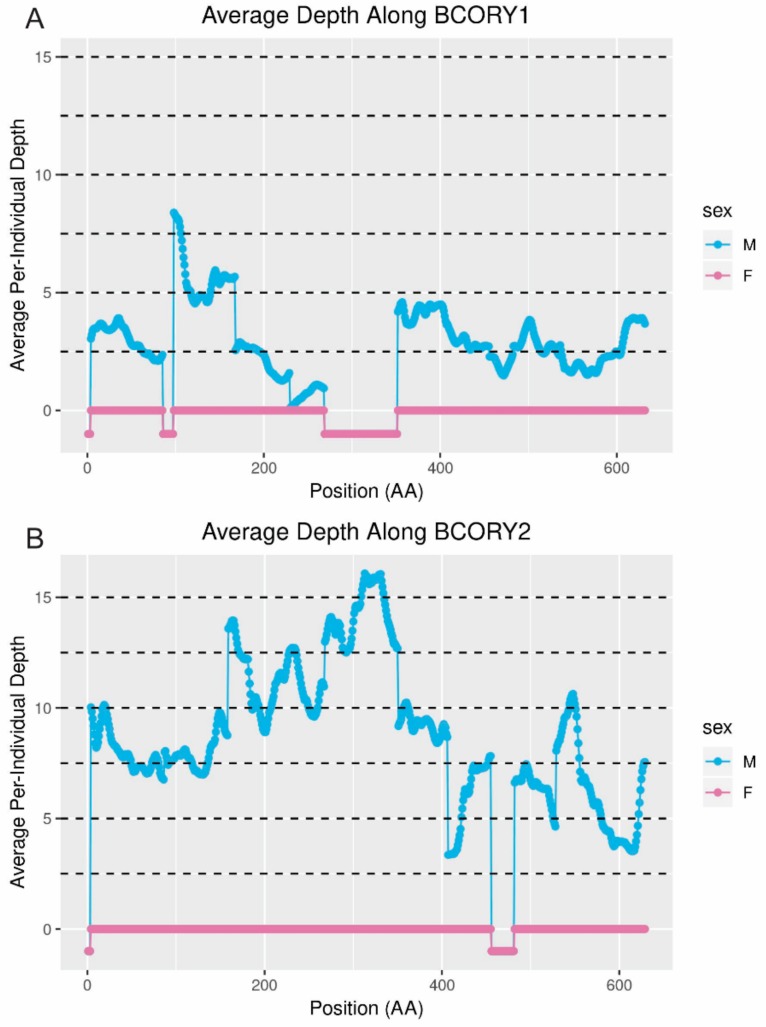
Estimated depth of coverage along *BCORY1* on scaffold360 (**A**) and *BCORY2* on scaffold310 (**B**). When the position of the codons could not be estimated from the BLAST results, the position is indicated below line at *y* = 0. Male and female depth is indicated separately. Each point represents an amino acid, with the depth estimated off of the surrounding 15 bp (approximately two codons on either side). Dotted lines indicate intervals of 2.5×, or approximately one copy.

In order to visualize depth in the exonic regions of the genes only, the genomic position of each dog exon in the fox genome was estimated using the BLAST output, and then the average depth over the nearest 15 bp was plotted, corresponding to the estimated position of the codon itself and two codons in either direction. In the case of fragmented genes, the best hit for each amino acid in the protein was used regardless of its scaffold of origin. Comparing *BCORY1* (assumed to be the copy on scaffold360) and *BCORY2* (assumed to be the copy on scaffold310) revealed that the increase in the depth of mapped reads occurred only on scaffold310 (Figure 6). While in some cases, the increased depth of coverage on *BCORY2* (scaffold310) was associated with a corresponding loss of coverage on scaffold360 (e.g., near the 300th amino acid), in general, this was not the case. 

**Figure 7:**
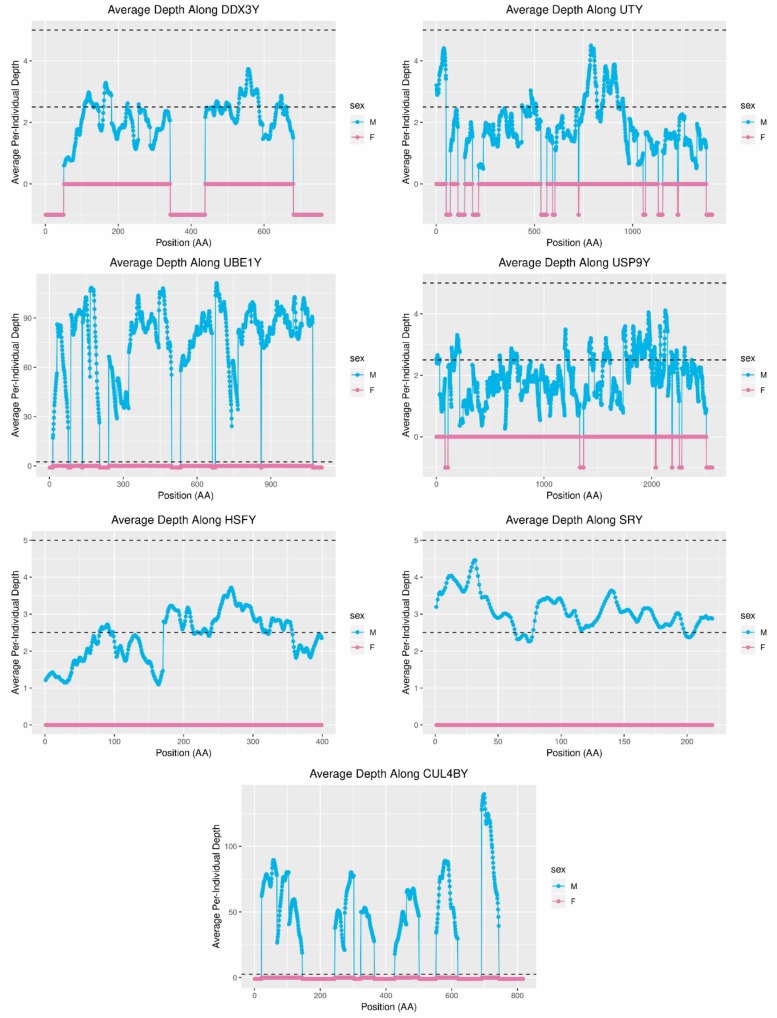
Depth of coverage in the predicted exons of MSY genes. Based on research in dogs and cats [6], *DDX3Y*, *USP9Y*, *UTY*, and *UBE1Y* (top two rows) were predicted to be single-copy in males, whereas *HSFY, SRY*, and *CUL4BY* (bottom two rows) were predicted to be multi-copy in males. The depth of coverage suggests a single copy (as indicated by the lower dotted line) for all of these genes, except *UBE1Y* and *CUL4BY*, which appeared to be present at a high copy number. When a codon’s position could not be estimated from the BLAST results, that region of the amino acid is indicated below 0.

The genes *DDX3Y, USP9Y, UTY*, and *UBE1Y* have previously been used as single-copy controls [6], so sequence depth was also calculated in the regions of the scaffolds corresponding to these genes and for *CUL4BY* and *SRY*—which are multi-copy in dogs and cats—and *HSFY*, which is multi-copy in cats but single-copy in dogs and wolves [6]. In most cases, the observed coverage of the single-copy genes was consistent with what was predicted. *DDX3Y*, *UTY*, and *USP9Y* all showed an average per-individual depth of approximately 0 in females and approximately 2.5 in males (Figure 7). However, *UBE1Y* showed a very high sequence depth that was consistent with up to 36 copies. In contrast, two of the three genes predicted to be multi-copy based on dog and cat showed a sequence depth more similar to the single-copy genes *DDX3Y*, *UTY*, and *USP9Y* (Figure 7). These results suggest that the red fox MSY may contain segmental and gene replications that differ from those of other carnivores.

## 4. Discussion

Although the red fox reference assembly was developed with short-read Illumina sequencing technology, we demonstrate here that the data produced by the project [27] is sufficient for the in silico identification of Y-chromosome sequence. The red fox genome project sequenced a male donor at 94× to assemble a 2.2-Gbp genome organized in 676,878 scaffolds and then sequenced an additional 30 foxes (15 male and 15 female) at approximately 2.5× per individual. Together, these resources made it possible to identify 171 scaffolds in the assembly constituting at least 1.7 Mbp of likely MSY sequence and containing 24 genes found on the Y-chromosomes of other carnivores.

Traditionally, Y-chromosome assembly projects targeting eutherian mammals have either flow-sorted chromosomes or used targeted BAC clones to amplify Y-chromosome sequence in vitro [6,8,10,63]. Such studies have also typically used long-read Sanger technologies for sequencing. While the emergence of affordable long-read next- and third-generation sequencing technologies is expected to benefit projects seeking to develop Y-chromosome assemblies for non-traditional mammalian models, analyses of species such as the polar bear [13], wolf [14], and now the red fox provide evidence that short-read technologies can also be leveraged for bioinformatic Y-chromosome sequence identification. 

Previous studies have differentiated potential Y sequence based on the ratio of female and male sequences mapping to a sequence fragment [11,13,64]. However, the assumption that female reads will not map to male-specific sequences is not always robust to misassembly in the reference genome [65]. Additionally, the presence of highly amplified Y-chromosome sequence can confound efforts to use sex differences in sequence coverage to identify likely Y-scaffolds. In the present analysis, in order to reduce the effects of these potential sources of error, we included a second metric to assess Y-chromosome specificity by deconstructing the scaffolds into 18-bp sequence motifs (18-mers) that were counted in the male and female resequenced reads. All the same, the disproportionate influence of sequencing depth on cluster assignment means that Y-scaffolds containing male-specific sequence motifs but showing similar depth of coverage across males and females may not have been detected in the present analysis (Appendix B). This limitation means that novel ampliconic or multicopy Y-chromosome genes could remain undetected in the red fox, and therefore future efforts to characterize the gene content of the red fox Y-chromosome should utilize approaches that do not rely on differences in depth of sequence coverage (e.g., as in the analyses conducted by [7]).

In the present analysis, two methods were used in conjunction to differentiate scaffolds likely to belong to different types of chromosomes. Unsupervised learning revealed three clusters consistent with the Y-chromosome, autosomes, and X-chromosome, with windows from 176 distinct scaffolds assigned to the cluster consistent with the Y-chromosome. This clustering method was very effective in identifying thresholds that discriminated different types of chromosomes without requiring empirical threshold adjustment, in contrast to other approaches [64,65]. Only 1.6% of windows assigned to the Y-chromosome cluster by the algorithm were ultimately removed during quality control, and assignments to all three clusters were in almost complete agreement with previous synteny-based analyses [27,28,41] (Figure 4). Re-assembly of the reads mapping to the putative Y-scaffolds also recapitulated the sequence content of the scaffolds themselves (Figure S1). The results therefore indicate that this multi-pronged approach worked to select only those scaffolds most likely to contain Y-chromosome sequence.

Research in well-developed mammalian genomic models such as dog and cat [6,7,35] facilitated the identification of red fox orthologs of carnivore MSY genes. Out of 18 dog MSY genes analyzed, 17 were identified in putative Y-linked red fox scaffolds, including all 11 of the core carnivore MSY genes [31]. The only dog protein that was not assigned a position in the fox scaffolds was *TSPY*. *TSPY* is X-degenerate, but BLAST analysis of the dog protein sequence against the red fox genome failed to identify any scaffolds meeting all mapping criteria, including an X-chromosomal copy (Table S1). This result is notable because *TSPY* is one of six genes found, either active or as a pseudogene, across the full range of eutherian mammalian taxa [6,44]. The analysis of MSY genes in the short-read-assembled polar bear reference genome also failed to identify a location for this gene [13]. Given that assumptions of parsimony would be violated by a loss of this gene in both red foxes and polar bears given its presence on the dog Y-chromosome, the more likely explanation is that multicopy, X-degenerate genes such as *TSPY* are particularly difficult to assemble from short-read sequencing. Despite the limitations preventing the identification of *TSPY*, these findings suggest that the gene content of the red fox MSY is very similar to that of the dog. Though this result is not surprising given the relatively recent divergence of dog and fox 6 to 9 million years ago, the human and chimp Y-chromosomes show significant divergence despite a similar estimated divergence date of 6 MYA [66].

However, there are also some ways in which the genes identified on the red fox MSY differ from those reported in dog. This study independently verified the presence of a Y-chromosomal paralog of *WWC3*, previously reported only on the wolf MSY [14]. *WWC3Y* was present in the predicted annotation of red fox scaffold310. Analysis of depth of sequencing near the X-chromosomal gene *WWC3* indicated that it is located near the pseudoautosomal boundary but also supported the existence of a male-specific copy (Figure 1). Lack of a strict pseudoautosomal boundary could explain for how this gene arose on the MSY in some canids and suggests that it could constitute a potential region of interest for studies of genetic diversity on the canid sex chromosomes [67]. However, another predicted gene on the Y-linked scaffold292 was not homologous to any known carnivore MSY genes. This prediction based on *RPS20*, a gene found on dog chromosome 20. *RPS4Y*, a gene in the same gene family found on the cat MSY, mapped to a position less than 400 Kbp away on the same scaffold. However, the nucleotide sequence of the predicted gene is very similar to a region of the dog MSY assembly [6]. Whether the gene is an artifact of annotation or a functional gene in the red fox is currently unknown. 

Additionally, two of four genes found on the cat but not the dog MSY, including *RPS4Y*, were identified in the red fox Y-scaffolds. This finding suggests that *AMELY* and *RPS4Y* may have been lost in dogs subsequent to divergence from red foxes 6 to 9 million years ago. However, using the same BLAST criteria as described for the cat–fox comparison to compare cat gene sequences to the wolf reference genome [42] revealed that one exon of *AMELY* does map to a Y-linked wolf scaffold [14] (Table S2). This result suggests that the loss of *AMELY* in dogs may be very recent.

Aggregation of gene content analyses across multiple taxa can facilitate estimation of the timing of gene loss and gain within phylogenies [31,38]. Thus, analysis of the red fox MSY revealed additional insight into the timing of gene loss, and possibly gain, among carnivores, though additional analysis would allow for timescales to be estimated more precisely (Table 6). Depth of coverage over MSY genes also offered insight into the timing of gene replication events in carnivores. Several copies of *SRY* are present in dogs and wolves [6,14], even though a single copy of this gene is more common across the mammalian phylogeny [38]. In foxes, *SRY* mapped to a single position in the red fox genome on scaffold431, and the depth of coverage in this region was consistent with one to two copies (Figure 7). This finding suggests that replication of this gene, which is critical to sex determination, occurred recently in the dog/wolf lineage. In contrast, depth of sequencing suggested that *UBE1Y*, which has been reported to be single copy in cats and dogs [6,38], may exist at as many as 36 copies in foxes. Interestingly, coverage of this gene in the grey wolf is consistent with two copies [14] and in the horse is consistent with at least eight copies [32,44]. *UBE1Y* is expressed testis specifically in mice and horses [32,68] and has been hypothesized to play a role in male fertility via germ cell proliferation [32]. Given that *UBE1Y* has been reported to have a higher rate of evolution in carnivores than other mammalian clades [31], further investigation into the phenotypic effects of its apparent replication in red foxes and/or wolves may be of interest. Present evidence of the gene content of the Y-chromosomes of species in *Pegasoferae* (i.e., the clade containing odd-toed ungulates, bats, and carnivores [69,70,71]) (Table 6) suggests that some genes, such as *AP1S2Y*, *RPS4Y*, *TMSB4Y*, and *WWC3Y*, are either present in more species than has currently been ascertained, or have undergone multiple gain/loss events within Carnivora. 

Although analysis of they-linked scaffolds in the de novo wolf assembly highlighted the possibility for X-degenerate genes and their Y-paralogs to be collapsed during assembly from short sequencing reads [14], in foxes, this type of collapsing was observed only for putative segmental replications. Patterns of misassembly commonly caused by the algorithm used for assembly from short reads explains why 3.4 Mbp of sequence was provided to ABySS, but only 1.7 Mbp was assembled: when constructing long sequences from short reads, the assembler must determine whether two sequences that are close, but not exact, matches belong in different places (e.g., repetitive elements or segmental duplications) or the same place (e.g., heterozygosity or sequencing errors). Patterns of depth of coverage over the Y-scaffolds in males and females suggested that male-specific repeats are likely to be collapsed, especially on the shorter scaffolds. The increasing feasibility of incorporating long-read next-generation sequencing into projects such as this will allow for more accurate resolution of highly repetitive genomic regions such as the Y-chromosome, even in non-traditional models and wildlife species.

In addition to the length of the assembly, short-read assembly can also influence the structure of sequence content within the assembly. Short scaffolds may be sequences that were erroneously excluded from larger contigs and scaffolds, and others may be orphaned by the collapsing of repetitive regions [3,4,5]. For example, *UBE1Y* and *CUL4BY*, which are likely to be ampliconic in the red fox genome (Figure 7; Table 6), were fragmented across several scaffolds, suggesting heterogeneity across copies may have resulted in scaffold breakage [2]. 

The opposite may have occurred on scaffold310: although, unlike in wolf, the genes *BCORY1* and *BCORY2* were assembled separately in the red fox scaffolds, depth in the region of scaffold310 containing *BCORY2* suggests that segmental replications were collapsed in this region of the assembly. A segmental replication in the region of *BCORY2* (Figure 7) is consistent with previous findings that microsatellite markers in this region can carry up to three alleles per male [28]. The presence of a segmental replication of this region was supported by sequence coverage of the nearby gene *ZFY* (Figure S2), which contains a microsatellite marker observed to carry up to two alleles per male [28]. However, the fact that multi-allelic Y-chromosome microsatellite markers were observed only in males from the North American subspecies of red fox (*Vulpes vulpes fulva*) suggests that these segmental replications, including additional copies of *BCORY2* and *ZFY*, may have evolved on a short timescale and may distinguish European and North American red foxes. Similarly, rapid changes in segmental replications have been reported in grey wolf populations and haplogroups [14].

With the red fox adding to the recent increase in the availability of comparative carnivore Y-chromosome resources, studies examining the timing and effect of Y-chromosome evolution, including replication events and gene loss and gain, have become increasingly feasible. The red fox sequence information presented here serves to refine the timing of differences in the Y-chromosome sequences of dogs and cats and also provides an outgroup for studies of Y-chromosome evolution during the evolution of dogs from wolves. These results support the set of Y-chromosome markers available for the red fox [18,28] and provide sequence that can be used for the development of additional tools for studying the evolution of the red fox Y-chromosome at a higher resolution and over longer timescales.

## 5. Conclusions

Sequence from the Y-chromosome was identified among the scaffolds comprising the short-read-assembled red fox draft reference genome. These scaffolds contained all 11 of the core Y-chromosome genes, as well as all but one of the genes previously identified on the dog MSY. The red fox Y-chromosome shows evidence of recent segmental replication that have resulted in an increase in copy number for the gene *BCORY2* and *ZFY*. The red fox also appears to carry many copies of the gene *UBE1Y*, and findings suggest that replication of *SRY* in the dog and wolf occurred subsequent to divergence with the fox 6–9 million years ago. This work demonstrates the potential for information pertinent to Y-chromosome tool development to be extracted through the bioinformatic analysis of even a highly fragmented draft genome, and supports previous findings suggesting recent segmental replications occurred on the Y-chromosome of North American foxes [28]. These genomic resources will facilitate the continued advancement of tools for studying red fox Y-chromosome diversity and the carnivore Y-chromosome more broadly.

## Figures and Tables

**Table 2:** Wolf MSY genes compared to the red fox genome. When dog X-chromosomal protein sequences were available, they were compared to the red fox draft genome using translated BLAST (tblastn). In the remaining cases, the sequences of the regions of the wolf reference genome [42] that contained the MSY genes [14] were compared to the draft red fox genome using standard nucleotide BLAST (blastn). Other than *DYNG*, which was initially reported in dogs [6], these genes have not been identified on the Y-chromosome of dogs or cats. NCBI refers to the databases maintained by the National Center for Biotechnology Information.

Gene	Source	Chromosome of Sequence Probe	Sequence Used
*AP1S2Y*	NCBI dog protein	ChrX	XP_005641196
*DYNG*	Wolf reference sequence	Y-linked scaffold	scaffold_2411:21000-65000
*TMSB4Y*	Wolf reference sequence	Y-linked scaffold	scaffold_3047:10000-13000
*WWC3Y*	NCBI dog protein	ChrX	XP_548855.3

**Table 3:** Positions of carnivore MSY genes on the red fox scaffolds. The approximate positions of each gene in the red fox genome was determined with BLAST.

Gene Name	Status	Scaffold #	Approximate Position Range	
*AMELY*	Present	549	18,297	26,696
*AP1S2*	Present	372	67,637	77,342
*BCORY2*	Present	310	7152	38,910
*BCORY1*	Present	360	112,755	137,091
				
*CUL4BY*	Fragmented	653	15,446	15,604
		2441	2922	3038
		2986	5148	5270
		948	15,791	15,883
		931	6211	6294
		573	6191	14,432
		2407	5919	6035
		367	113,983	124,732
				
*DYNG*	Present	367	25,206	66,258
*DDX3Y*	Present	292	203,960	210,475
*EIF1AY*	Present	292	596,932	597,039
*EIF2S3Y*	Present	310	135,297	168,226
*FLJ36031Y*	Not found	-	-	-
*HSFY*	Present	292	647,018	648,668
*KDM5D*	Present	292	486,476	499,109
				
*OFD1*	Fragmented	758	9373	9579
		732	3168	2995
				
*RBMYL*	Present	444	13,493	22,483
*RPS4Y*	Present	292	636,415	636,457
*SRY*	Present	431	74,758	75,417
*TETY1*	Not found	-	-	-
*TETY2*	Present	292	6025	6288
*TMSB4Y*	Present	372	50,666	53,690
*TSPY*	Not found	-	-	-
				
*TXLNGY*	Fragmented	444	50,789	50,884
		360	8239	21,762
				
*UBE1Y*	Fragmented	504	28,869	42,400
		612	22,935	23,670
		703	16,522	25,924
		828	5048	14,951
		1224	6911	7033
				
*USP9Y*	Present	292	238,257	355,974
*UTY*	Present	292	39,840	178,019
*WWC3Y*	Present	310	303,473	425,370
*ZFY*	Present	310	55,006	67,293

**Table 4:** Dog orthologs of red fox predicted genes. The best-hit canine protein sequence was selected using protein BLAST (blastp) against NCBI protein database. When the best-hit gene is not located on the dog Y-chromosome, its chromosomal position is also indicated. The overlap column describes whether the position of the predicted gene in the red fox genome overlaps the position assigned using the dog version of the gene as a query against the fox genome.

Predicted Gene	Scaffold	Start	End	Dog Best Hit Name	Accession Number	Overlap
Vulp_V011272	292	28,248	178,019	UTY	NM_001284484.1	Yes
Vulp_V011273	292	185,825	186,022	RPS20 (chr20)	XM_542128.5	No
Vulp_V011274	292	202,921	215,729	DDX3Y	JX964855.1	Yes
Vulp_V011275	292	236,816	355,974	USP9Y	JX964851.1	Yes
Vulp_V011276	292	466,274	499,306	KDM5D	NM_001113458.1	Yes
Vulp_V011277	292	647,015	648,668	HSFY	JX964870.1	Yes
Vulp_V012194	310	54,922	67,302	ZFY	JX964866.1	Yes
Vulp_V012195	310	165,975	168,466	EIF2S3 (chrX)	XM_537983.6	Yes
Vulp_V012196	310	218,169	450,681	WWC3 (chrX)	XM_548855.6	Yes
Vulp_V014417	431	74,746	75,342	SRY	KP081776.1	Yes
Vulp_V018159	703	14,088	19,601	UBE1Y	JX964860.1	Yes

**Table 5:** Clusters identified with *k*-means clustering. The centers of the clusters identified by *k*-means clustering are based on a Cartesian plot of the two metrics. The chromosome type most consistent with each cluster’s position is indicated. As expected, because the scores are normalized, the centroid for the autosomes is at approximately (0,0), whereas the centroid for the X-chromosome is to the left of 0 (i.e., the normalized CNV-Seq score is negative), and the centroid for the Y-chromosome is to the right (i.e., the normalized CNV-Seq score is positive).

Cluster	Normalized Percent Mapped Reads Contributed by Males (CNV-Seq)	Normalized Percent 18-mers Unique to Males (YGS.pl)	Windows	Scaffolds	Most Likely Chromosome Type
1	11.173	1.178	752	180	Y-Chromosome
2	0.173	−0.326	461,795	11,263	Autosome
3	−3.822	−0.399	23,200	305	X-Chromosome

**Table 6:** Summary of gene status in horse, cat, grey wolf, dog, and red fox based on the current literature. Grey shading indicates genes for which copy number estimates were available in all five taxa, and an estimated copy number is provided in these cells. Wolf estimates for *AMELY, FLJ36031Y*, and *RPS4Y* were determined by BLAST against the wolf reference genome using the same parameters used in the fox versus cat analysis (Table S2). Starred gene names (*) indicate the 11 core carnivore Y-chromosome genes [31]. +/- indicate binary presence/absence of gene based on current literature. ++ indicates genes that are multicopy but with no estimate of copy number specified. The question mark (?) indicates uncertainty about gene status. +* indicates ambiguity in the literature about which copy of a duplicated gene was observed.

Gene/Gene Family	Horse	Cat	Red Fox	Wolf	Dog
*AMELY*	+	+	+	?	-
*AP1S2Y*	+	-	+	+	-
*BCORY1*	+*	-	+	+*	+
*BCORY2*		-	+		+
CUL4BY *	9	3+	~25	~10	++
TXLNGY *	+	+	+	+	+
DDX3Y *	+	+	+	+	+
*DYNG*	-	-	+	+	+
EIF1AY *	+	+	+	+	+
EIF2S3Y *	+	+	+	+	+
*FLJ36031Y*	-	+	-	-	-
*HSFY*	3	11	1	1	~1
KDM5D *	+	+	+	+	+
*OFD1*	+	+	+	+	+
RBMYL *	+	+	+	+	+
*RPS4Y*	-	+	+	-	-
SRY *	1	~4	1	3	7
*TETY1*	-	+	-	+*	-
*TETY2*	-	+	+		+
*TMSB4Y*	+	-	+	+	-
*TSPY*	13	10	-	~100	25+
UBE1Y/UBA1Y *	8+	1	~36	2	1
USP9Y *	+	+	+	+	+
UTY *	+	+	+	+	+
*WWC3Y*	+	-	+	+	-
*ZFY*	+	+	+	+	+
References	[32,44]	[6,7,31,35,44,72]	Table 2 and Table 3	[14]; Table S2	[6,7,44]

**Figure A1:**
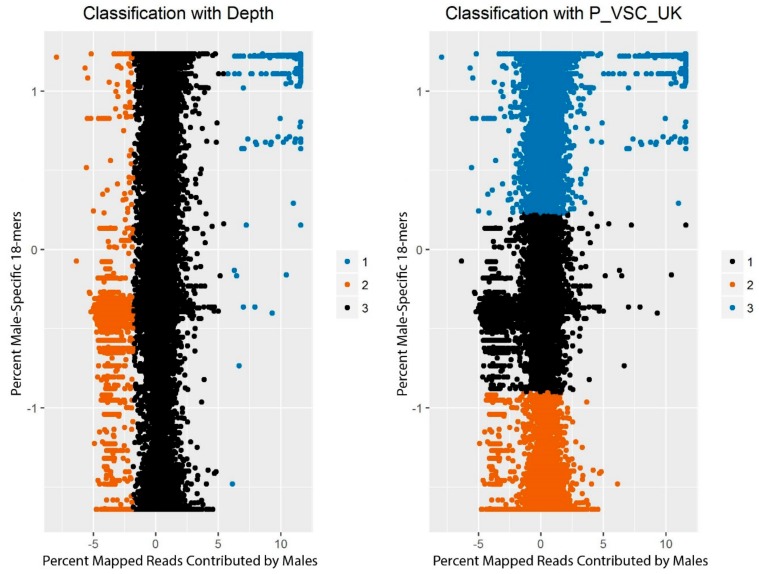
Cluster assignment of windows based on each metric individually. Cluster number is random, but clusters are color-coded to indicate whether they are most consistent with the X-chromosome (orange), autosomes (black), or Y-chromosome (blue).

**Figure A2:**
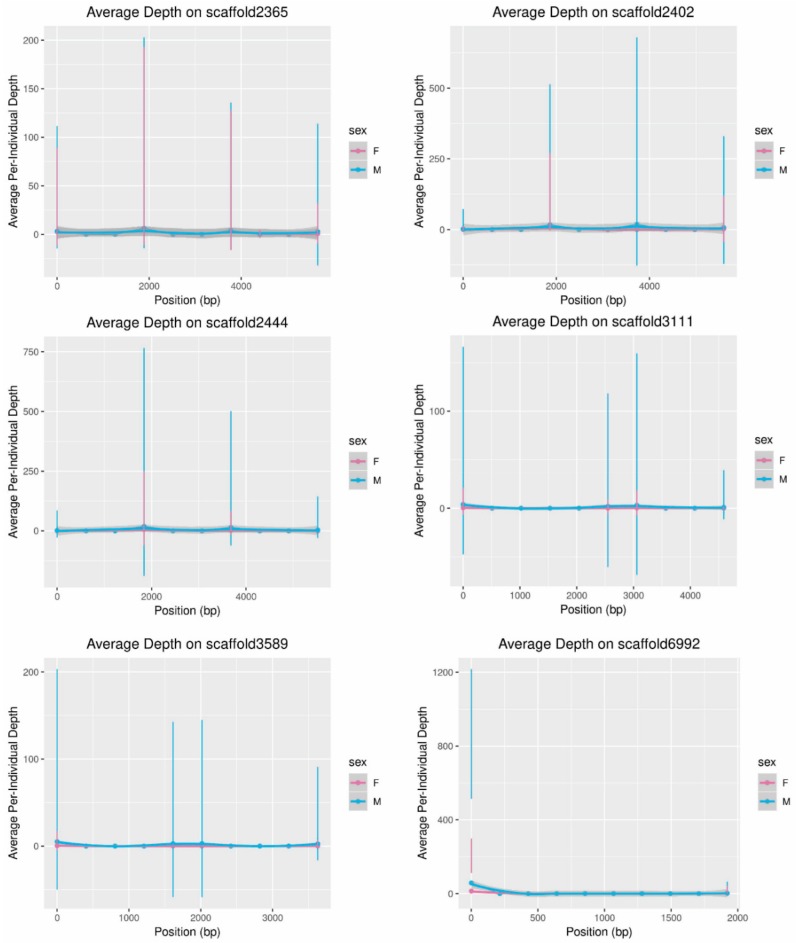
Depth of coverage of scaffolds included in cluster 1 that were assigned to an autosome based on P_VSC_UK alone. Depth in male and female resequencing reads is indicated separately. Depth was averaged in males and females in windows of 10% of the scaffold’s length. Axes vary depending on the observed depth (*y*) and the length of the scaffold (*x*).

## Supplementary Materials

The following are available online at https://www.mdpi.com/2073-4425/10/6/409/s1, Figure S1: MashMap visualization of the ABySS assembly aligned to the Y-scaffolds from the red fox reference genome; Figure S2: Estimated depth of coverage of the exons of gene *ZFY* on scaffold310; Table S1: Locations of X-chromosomal and autosomal paralogs of MSY genes; Table S2: BLAST results of three cat MSY genes against the wolf reference genome. Table S3: Scaffolds identified as containing Y-chromosome sequence; Table S4: Scaffolds constructed with ABySS from reads mapping to putative Y-scaffolds in the red fox assembly. The original BLAST outputs and Y-scaffold fasta sequence, and ABySS assemblies have been deposited in the Illinois Data Bank at https://doi.org/10.13012/B2IDB-4447017_V1.

## Appendix A

In order to disambiguate the *BCOR*-like genes on scaffold310 or scaffold360, the region identified on each scaffold with BLAST (Table 3) as most likely to contain the gene sequence was extracted from the scaffolds and converted to predicted peptides using GENSCAN [73,74]. Next, the predicted peptide was used as a query against the NCBI protein database using protein BLAST (blastp) through the BLAST web interface. The dog gene with the highest percent identity was then selected.

For scaffold310, the region scaffold310: 0–48910 bp was extracted and provided to GENSCAN. The best dog protein hit was *BCORY2* (AGS47770.1), with total score of 1181, an e-value of 0.0, and a percent identity of 85.65%. The predicted peptide was: XSGVYQMDGSVDVLPALQSKNPSPLVVKNNPEQPGWLSGLAPALAQGEDTAVKTYSLFKAPEDKNLPVKKYFMDRWPMNKPPAMDILHGPTLRLDRKHKLSGNRTDTETTVEETPEDPLQKAKQRRTSKGLHPKKQQQLLHFRKRWEQQVSAEESKPGQKSGKEMAQEVQTDVTAQENSCSKEKPHREGAEAKTNRSLSEETFKSSDHEQGFPIFSTSLPVKSLLSTTISTLPASSLHSKLQKIKESQKRHVLCTDEKDHQAASVLQKYTKNSEKPSGKRLCKTKHLISHESRQDLVVPADNSVGYDDGKVTIGRVKKQERPRSKYYVPPGNWDEKPISRLQQILPASQSSQLLHSRSPPETTQSQSMLPEAQRPMVRNAGETLLQRAAQLGYEELVLYCLENNICDVNHQDNAGYSALHEACAGGWLHIAQHLLEYGADVNRSAHDGTRPLHDAVENDHLEIVRLLLSYGADPTLATYSGRTIMKMTHSELMKMFLAGSKNTQIISHIERDVKDRSSNIIANDGTLACDCTDIKPHPHLGGHQTIQQSFLNHYLSDLRGHSDEFIAPWEFYGSSVCEPDKKAGHNVLANRPGPEDQDDEDKANNSDVFEFSDSPLLPWYNIQVSVCQRARKWFLLSDVLKKLEMSPCIFRCNFPNIEIITISEAEFYRQVSASFLYSCSKELDAFNPESKELLDLVEFTNELQTLLGSSMKLLNPSDVALEKDH.

Likewise, for scaffold360, the region scaffold360: 102755–147091 was extracted. The dog protein best hit was *BCORY1* (AGS47779.1) with a total score of 1061, an e-value of 0.0, and an identity of 66.60%. The predicted peptide of this copy was: MLGLRGRGPRRGWAARVALPSGAGLLAAGPGEAGLGCKRLRPALGGAVVPRPAWLPFVAGPPGAGPHRQEPSPRSSLAFGCTRPRKVLTGPRSPWRLRATRSSEPRAPLTVCGEDSAVKKDSLLKAPKDKNLPVKKYFMNRWPMNKSSAMNILNNPTLQLDRKRKLSGNSTDTEITVDETPEDPLQKAKQGWTSKGLHPKKQQQLLHFRKRWEQQVSAEESKPGQKSGKEMAQEVQTDVTAQENSCSKEKPHREGAEAKTNRSLSEETFKSSDHEQGFPIFCTALPVKSLLSTGICTLPASSLHSKLQKIKESQKTHVLCTDEKDHQAASVLQKYTKNSEKPSGKRLCKTKHLISHESRQDLVVPADNSVGYDDNKIVPFKSGLYRLITHSLLASEKPSGKRLCKTKHLISHESRQDLVVPADNSVGYDDNKELVLYCLENNICDVNHQDNAGYSALHEACAGGWLHIAQHLLEYGADVNRSAHDGTRSNPALYQAAVYVFLELYQSTSKHYGGYRNIYTDTGNLLIAASLHGGLSPILSLFFCMLQEIAPVSARGCTVFLALFIEDFLFSIGYSFPLCRGERERGAETQTEGEAGSMHREPDVGLDPGTPGLCPGPKAGAKLLRHPGIPCRPLHDAVENDHLEIVRLLLSYGADPTLATYSGRTIMNMTHSELMKMFLADYFSDLRGHSDDEFIAPWEFYGSSVCEPDEKAGHNVLANPPGPEDQDDEDKANNSDMFEFEFSDSPLLPCYNIQVSVCQRARNWFLLSDVLKKLKMSSCIFRCNFPNIEIITISEAEFYRQVSASFLYSCSKELDAFNPESKELLDLVEFTNELQTLLGSSMELLNPSDMALEKDD.

## Appendix B

In order to evaluate the role of each of the two metrics (the frequency of male-specific 18-mers and the ratio of male to female reads mapping in each window) in clustering outcomes, *k*-means clustering was also run using the same parameters (nstart = 100, centers = 3) on the normalized CNV-Seq and YGS.pl outputs individually (Figure A1).

Comparing the cluster assignment for each window using each of the two metrics separately revealed 36,696 windows that were assigned to the Y-chromosome by at least one metric. Only nine of these windows were ultimately placed in cluster 1, corresponding to the Y-chromosome, in the combined *k*-means clustering analysis (as described in Materials and Methods). Moreover, in all nine cases, P_VSC_UK was in disagreement with the ultimate assignment of the window. In only three cases did the analysis of the CNV-Seq data alone assign a window to the Y-chromosome when YGS.pl and the joint analysis assigned it to an autosome or the X-chromosome. This phenomenon was likely based on the fact that there were so many more autosomal windows than windows from the allosomes, especially the Y-chromosome, and that many autosomal windows contained a high proportion of 18-mers that were unmatched in the female resequencing data.

**Table A1:** Variation in window assignment under different clustering parameters. The combined analysis was conducted using both metrics. Although cluster number is arbitrary, the most likely chromosome type is indicated for each cluster based on the predicted distribution of scores among the X-chromosome, autosomes, and Y-chromosome. Defining clusters using each metric alone revealed that 36,696 windows were assigned to a Y-chromosome cluster based on at least one metric. Grey shading indicates cells where the most likely chromosome type matched between a single metric and the combined clustering analysis.

			CNV-Seq Only			YGS.pl Only		
			1	2	3	1	2	3
			*Y*	*X*	*Autosome*	*Autosome*	*X*	*Y*
**Combined**	1	*Y*	9	0	0	9	0	0
	2	*Autosome*	0	1	13,892	1	5776	8116
	3	*X*	0	22,792	2	22,792	2	0

Based on these observations, the windows assigned to cluster 1 (Y-chromosome) in the combined clustering analysis were examined. In this group of nine windows, two came from a scaffold known to map to the X-chromosome [41], and 487 out of their combined 495 windows were ultimately assigned to cluster 3 (consistent with the X-chromosome) in the joint analysis. Therefore, these were windows that had been excluded prior to assembly with ABySS. The other seven windows were from scaffolds ranging in size from 2133 to 6292. Because these scaffolds were broken up into only one or two windows in the CNV-Seq analysis, heterogeneity in depth along the scaffold (e.g., due to presence a repetitive element found on the Y-chromosome that was missed during repeat masking) could artificially inflate the apparent copy number of the scaffold (Figure A2).

Based on this analysis, the P_VSC_UK scores of the scaffolds assigned to cluster 1 in the joint clustering analysis were examined, and those with scores lower than 65% were discarded from downstream analysis. Scoring the scaffolds for P_VSC_UK using YGS.pl therefore provided data that complemented the depth-based analysis. Ultimately there were too many male-specific *k*-mers in the dataset as a whole for the metric to separate Y-specific sequences as effectively as it did in drosophila and humans [36]. This result may also speak to the fact that short scaffolds, even up to 10 Kbp, in short-read-sequenced de novo assemblies are in many cases orphan sequences resulting from the misassembly of repetitive regions [5], as all of the scaffolds flagged with his metric were shorter than 7.5 Kbp.
