# Supplementary figures and images for: The Red Fox Y-Chromosome in Comparative Context
Source: Genes (Basel). 2019 May 28;10(6):409. doi: 10.3390/genes10060409 (PMC6627929; doi:10.3390/genes10060409)

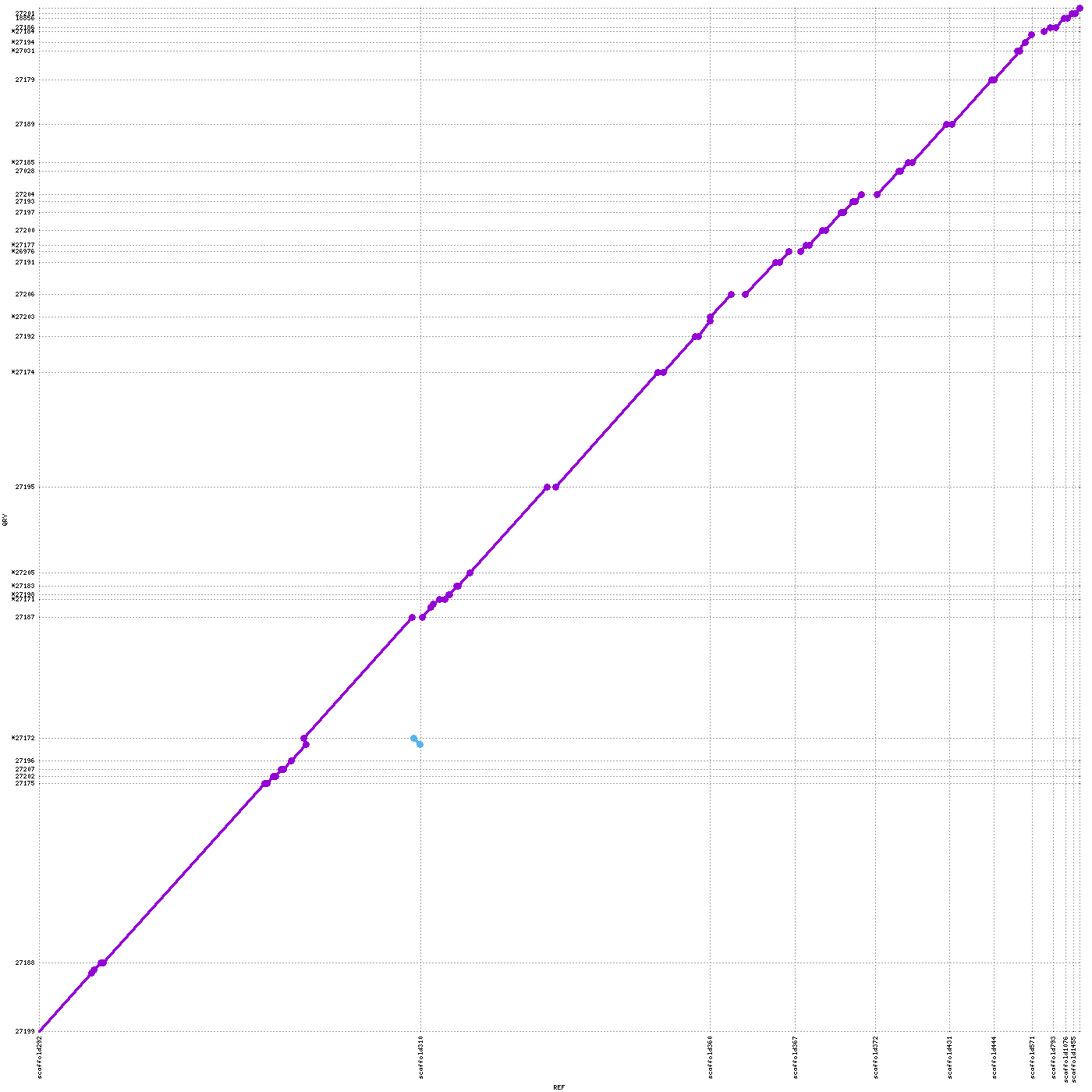

Supplement: Supplementary file 1 [file genes-10-00409-s001.zip › FigureS1. MashMap Output.png]

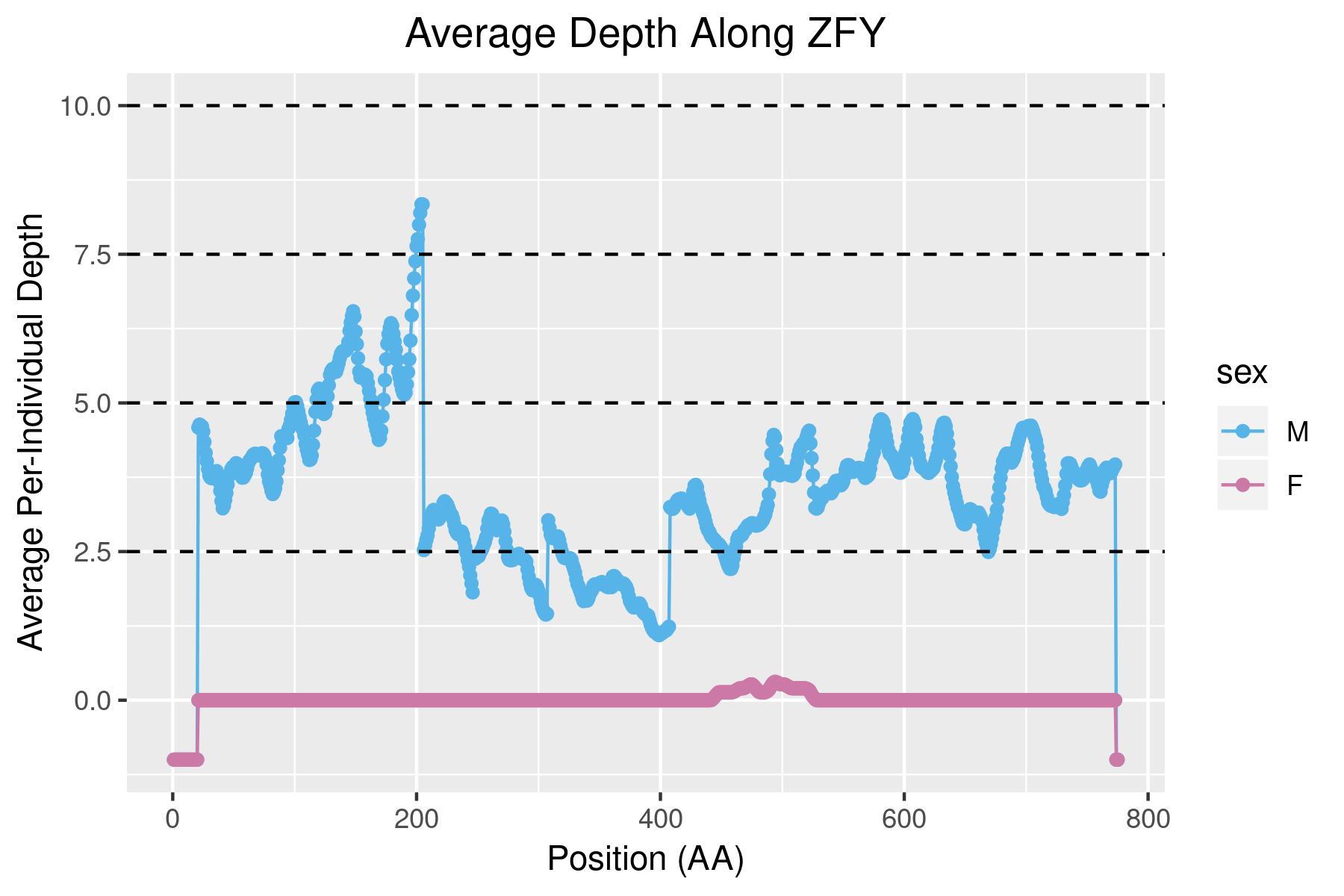

Supplement: Supplementary file 1 [file genes-10-00409-s001.zip › FigureS2. Coverage of ZFY.jpg]
